# Assessing the prevalence and severity of asthma, rhinitis, and eczema among schoolchildren (6–7 and 13–14 years old) in Khuzestan, Iran: a cross-sectional survey

**DOI:** 10.1186/s12887-022-03520-x

**Published:** 2022-08-02

**Authors:** Maryam Dastoorpoor, Narges Khodadadi, Farzan Madadizadeh, Hanieh Raji, Elham Shahidizadeh, Esmaeil Idani, Maryam Haddadzadeh Shoushtari

**Affiliations:** 1 Department of Biostatistics and Epidemiology, Air Pollution and Respiratory Diseases Research Center, Ahvaz Jundishapur University of Medical Sciences, Ahvaz, Iran; 2 Research Center of Prevention and Epidemiology of Non-Communicable Disease, Department of Biostatistics and Epidemiology, School of Public Health, Shahid Sadoughi University of Medical Sciences, Yazd, Iran; 3 Department of Internal Medicine, Air Pollution and Respiratory Diseases Research Center, Ahvaz Jundishapur University of Medical Sciences, Ahvaz, Iran; 4 Department of Environmental Health Engineering, Faculty of Medical Sciences, School of Health, Abadan, Iran; 5 Department of Internal Medicine, School of Medicine, Shahid Beheshti University of Medical Sciences, Tehran, Iran

**Keywords:** Allergies, Asthma, Rhinitis, Eczema, Prevalence, Severity

## Abstract

**Background:**

Asthma and allergic complications are the most common chronic disorders in children and adolescents. This study aimed to determine the prevalence and severity of asthma, allergic rhinitis, eczema among schoolchildren, and some related risk factors.

**Methods:**

The cross-sectional study was performed in 2019 and involved 4000 students aged 6–7 years and 4000 students aged 13–14 years (both girls and boys) from urban schools in Khuzestan Province, southwestern Iran. We used the multi-stage sampling method. Data were collected using the Persian version of the International Study of Asthma and Allergies in Childhood (ISAAC) questionnaire.

**Results:**

The prevalence of current wheeze, wheeze ever and asthma ever in the age group of 6–7 years was 3.8, 4.5, and 1.8%, respectively; in the age group of 13–14 years, it was 4.4, 5.9, and 3.4, respectively. In terms of gender, males (4.9, 6.0, and 2.7 percent, respectively) had substantially higher rates of current wheeze, wheeze ever, and asthma ever compared to the girls (2.8, 3.6, and 2.0 percent, respectively) (*p* < 0.001). The prevalence of rhinitis, Hay fever, and eczema among young people was 5.3%, 3.5%, and 1.0%, respectively. Current wheeze was more common in children with allergic rhinitis in the last 12 months (30.3% vs. 2.7%, *p* < 0.001), Hay fever (32.8% vs. 0.3%, *p* < 0.001) and eczema (27.8% vs 3.8%, *p* < 0.001), compared to children who did not.

**Conclusions:**

The prevalence and severity of asthma symptoms were significantly associated with allergic rhinitis, eczema, and gender.

**Supplementary Information:**

The online version contains supplementary material available at 10.1186/s12887-022-03520-x.

## Background

Allergic phenomena include various signs and symptoms. The allergic process emerges from an atopic disease with eczema and progression to asthma, leading to allergic rhinoconjunctivitis [1]. Eczema, a predisposing factor for asthma, is more likely to appear as a chronic inflammatory itchy skin rash in infancy and childhood [2, 3]. Asthma is a significant public health problem and children's most common inflammatory disorder. Extensive and variable airway obstruction due to inflammation causes mild to severe symptoms such as wheezing, shortness of breath, chest tightness, and frequent recurrent cough [4]. The most prominent clinical symptoms of allergic rhinitis are sneezing nasal congestion, and runny and itchy nose and eyes [5]. Although the exact root of allergic diseases is unknown, genetic and environmental factors such as air pollution, cold, smoking, mold, and pets are major stimulating factors [6].

Recent trends in respiratory medicine have emphasized patient symptoms and their impact on quality of life rather than solely on physiological evaluations [7]. Physicians use symptom-based questionnaires to diagnose and treat chronic airway diseases like asthma and allergic rhinitis [8]. The International Study of Asthma and Allergies in Childhood (ISAAC), (1991) provides reliable information on the incidence, symptoms, and changes over time and has been accepted as a standard questionnaire in worldwide epidemiological studies on asthma and allergies in children [9].

Over the past few years, the world has witnessed a significant increase in the prevalence of atopic diseases, especially in children in both developed and developing countries [10]. According to a systematic review of 50 studies in the Middle East, children under 18 were more likely to suffer from asthma (according to ISAAC criteria) than children aged 13–14 years or younger (7.47%); for children aged 6–7 years, it was 7.43% [9]). According to reports, the prevalence of asthma in Iran was 2.7% in school students aged 6–7 and 3.5% in students aged 13–14 [11]. According to different studies in Iran, allergic rhinitis is prevalent at 11–40% [12–14], while eczema is prevalent at 4.1–15% [13, 15, 16]. Allergic disorders put a heavy economic and social burden on the family and society. The symptoms of these disorders in children can result in challenging behavior, poor school performance, and reduced quality of life [17]. Therefore, early detection and management of these diseases in childhood reduce their complications, and a correct understanding of their true prevalence can help prevent, treat, and adequately reduce their prevalence.

Asthma is a complex illness influenced by genetic [18, 19], infectious [20], nutritional [21], socioeconomic [22], psychological [23], and environmental variables [24, 25]. Outdoor pollutants, including benzene, particles (such as PM_10_ and PM_2_._5_), and irritating gases (such as nitrogen oxide (NO_2_), ozone (O_3_), and sulfur dioxide (SO_2_)) enhance the occurrence of respiratory disorders, particularly asthma, in industrialized nations [26]. Over the years, Khuzestan Province has endured a variety of pollutants, including micro-waste, industrial, and non-industrial pollutants. The capital of Khuzestan Province is Ahvaz. Pollution levels in Ahvaz are rising and growing by the day [27, 28]. According to a 2013 World Health Organization report, Ahvaz is the most polluted city in the world in terms of the average yearly quantity of suspended particles fewer than 10 µm (372 g/ m3) [29]. Even though air pollution levels have increased due to industrial and non-industrial pollutants, there has been no study of the incidence of these illnesses in children in Khuzestan Province. This study aimed to use the ISAAC Questionnaire to estimate the prevalence of asthma, rhinitis, and eczema in 6–7-year-old and 13–14-year-old students in urban schools in Khuzestan Province and some related risk factors.

## Methods

### Place of study

**Fig. 1:**
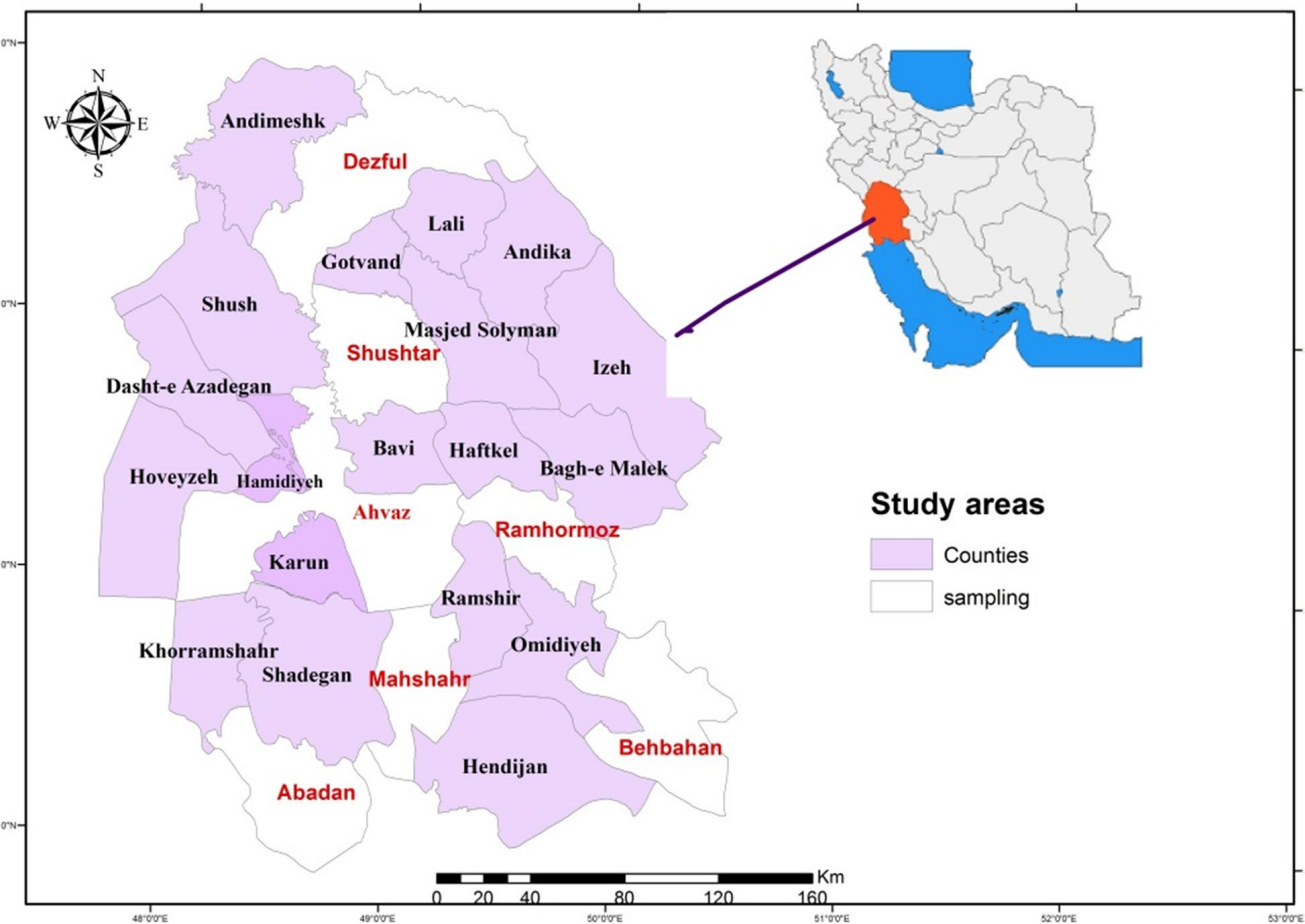
Location of Khuzestan Province in Iran and Sampling Counties

Khuzestan Province is located on the Persian Gulf coast in southwest Iran and is the center of Iran's oil and gas production. This province, with a land area of 64.055 square kilometers and a population of around 4.711 million people (according to the 2016 census), is Iran's fifth most populated province [30]. Khuzestan is at 31.33 degrees north latitude and 48.69 degrees east latitude (Fig. 1).

The population of students aged 6–7 years in the whole of Khuzestan province was 69,420, and the population of 13–14 students was 109,591. Through multi-stage sampling, this study examined the characteristics of 4000 students aged 6–7 years and 4000 students aged 13–14 in a Khuzestani public school and a private school.

In order to collect the data, questionnaires were developed based on the Phase 1 ISAAC questionnaire [31], which had previously been standardized and localized in Iranian studies [32].

The ISAAC questionnaire is a standard tool used in the International Study for Asthma and Allergy in Children. The questionnaire includes variables such as: gender, wheezing, history of wheezing in the last 12 months, number of possible attacks during the last 12 months, asthma, a history of runny nose and eyes during the last 12 months, post-exercise wheezing, dry cough at night during the last 12 months, dysfunction in daily routines, Hay fever, having eczema, a history of eczema during the last 12 months, the area affected by eczema, itching in sleep and the presence of eczema. The ISAAC self-administered questionnaire is the most common method widely used to determine the prevalence and severity of asthma and allergic diseases. We recommend using school-age children ages 6 to 7 and 13 to 14 for the questionnaire. The older age group was chosen to reflect the era when asthma mortality was more prevalent and to allow the use of a self-completed questionnaire. The age range of 6 to 7 years represents early infancy since this is when asthma attacks and hospitalizations occur most frequently [33].

The data collection checklist in this study included: age, sex, and some possible risk factors such as the family history of asthma and other allergic diseases, birth weight, breastfeeding, smoker in the household, and pets in the home.

### Sample size

In a 2012 study by Ghaffari et al. in Sari (Iran), the prevalence of pediatric asthma was 12%, the prevalence of rhinitis was 17%, and the prevalence of eczema was 6% [34]. Based on a prevalence of 6%, we calculated the sample size based on the lowest prevalence. We included about 4000 people in the final sample size under the design effect1.5 for each elementary and first grade. The sample volume formula is as follows:

$$\mathrm n=\frac{Z_{1-\frac\alpha2}^2\;p\left(1-p\right)}{d^2}=2675$$

$$\alpha =0.05\;{ Z}_{\begin{array}{c}\;1- \frac{ \alpha }{2}\end{array}}=1.96,\;p=0.06\;d=0.15p$$

### Sampling

The sampling process consisted of the following eight steps:

First, Khuzestan Province was divided into five geographical areas (north, south, east, west, and center). Then, we selected the most populous cities in each geographical area (7 cities were selected). Then, through multi-stage random sampling, the number of private and public schools (clusters) required for sampling from each city were identified by probability proportional to size cluster sampling (PPS-CS). A total of 200 clusters (100 primary schools and 100 secondary schools) were selected from 7 cities (by PPS-CS); then, in each school, 40 students were selected through the random sampling method (Supplementary 1).

### Analysis

The study used survey analysis. A survey analysis uses a sample structure that reflects the composition of the community and obtains its point estimates by comparing the sample to the actual population. This is also the case when proportional sampling is used. However, in situations where the response rate is not 100%, survey analysis might cancel the difference between the sample and target population.

The results were related to seven counties in the North, South, East, West, and Center, and the research tended to generalize it to all children aged 6–7 and 13–14 years living in Khuzestan Province. The research weighed samples from all cities. This indicates that each sample represents several people.

Three probabilities were multiplied and inverted to obtain the total weight for each sample.

- Probability of choosing any city in Khuzestan Province;
- Probability to select each cluster in each city; and
- Probability to select any school from any cluster.

The research used frequency indices to estimate the descriptive results. In addition, the researchers used the chi-square test to measure the analytical results and examine the relationship between dependent variables (asthma symptoms and allergic conditions) and independent variables (such as; age and sex). A multivariable logistic regression model was used to identify potential risk factors for current wheeze (wheezing in the past 12 months). Software Stata12was used to analyze, and a *p*-value below 0.05 was considered significant.

## Results

This study distributed 8000 questionnaires, and 7344 were returned, including 3355 girls and 3989 boys. The response rate was almost 92%. More than 39% of samples came from Khuzestan Province. Table 1 summarizes the demographic and clinical characteristics of the samples.

**Table 1:** Demographic and clinical characteristics of the sample (*n* = 7344)

Variable	Class	N	%	Weighted %^a^
Age in years	6–7 years	3681	50.1	62.7
	13–14 years	3663	49.9	37.3
Sex	Girl	3355	45.7	41.2
	Boy	3989	54.3	58.8
Ever had wheezing	No	6949	94.6	95.0
	Yes	395	5.4	5.0
Wheezing in the last 12 months (Current wheeze)	No	7045	95.9	96.0
	Yes	299	4.1	4.0
Asthma ever	No	7135	97.2	97.6
	Yes	209	2.8	2.4
Rhinitis ever	No	6904	94.0	94.7
	Yes	440	6.0	5.3
Rhinitis in the last 12 months	No	6961	94.8	95.3
	Yes	383	5.2	4.7
Hay fever ever	No	7033	95.8	96.5
	Yes	311	4.2	3.5
Eczema ever	No	7249	98.7	99.0
	Yes	95	1.3	1.0
Family history of asthma and other allergic diseases	No	6043	82.3	82.8
	Yes	1301	17.7	17.2
Birth weight	Normal	7109	96.8	97.5
	Low	235	3.2	2.5
Breast feeding	Breast milk	6226	84.8	85.9
	Milk Powder	314	4.3	3.8
	Both	804	10.9	10.3
Smoker in the household	No	6411	87.3	88.3
	Yes	933	12.7	11.7
Pets in the home	No	6280	85.5	88.4
	Yes	1064	14.5	11.6
Area of residence	North	1161	15.8	17.8
	South	1501	20.4	16.9
	East	520	7.1	5.0
	West	1320	18.0	11.4
	Center	2842	38.7	48.9

^a^ Estimates were weighted using the bootstrap weights

Table 2 presents the prevalence and severity of asthma, allergic rhinitis, and eczema symptoms in children aged 6–7 years and 13–14. The prevalence of current wheeze, wheeze ever, and asthma in the 6–7 age group was 3.8%, 4.5, and 1.8%, respectively. In the age group of 13–14 years, these were 4.4%, 5.9%, and 3.4%, respectively. The prevalence of current wheeze, ever wheeze, wheezing limited speech to 1 or 2 words, asthma ever, exercise-induced wheeze, and nocturnal cough was significantly more common in the age group of 13–14 years than in the age group of 6–7 years (Table 2).

**Table 2:** Weighted prevalence estimates for prevalence and severity of asthma, rhinitis, and eczema by age^a^

Asthma symptoms	6–7 years (*n* = 3681)	13–14 years (*n* = 3663)	*P*-Value^b^	
Ever had wheezing	**4.5**	**5.9**	** < 0.001**^**c**^	
Wheezing in the last 12 months	**3.8**	**4.4**	** < 0.001 **^**c**^	
Number of attacks of wheezing in the last 12 months	None	0.1	0.3	< 0.001 ^c^
	1–3 times	3.1	2.7	
	4–12 times	0.6	1.1	
	> 12 times	0.1	0.4	
Sleep disturbance due to wheezing in the last 12 months	None	1.3	1.7	< 0.001 ^c^
	< Once/week	1.7	1.8	
	> Once/week	0.7	0.9	
Wheezing limited speech to 1 or 2 words	1.2	1.5	< 0.001 ^c^	
Asthma ever	**1.8**	**3.4**	** < 0.001 **^**c**^	
Wheezing occurring during or after exercise	2.2	4.4	< 0.001 ^c^	
Dry night cough at night unrelated to cold/chest infection	4.0	6.7	< 0.001 ^c^	
Rhinitis ever	4.6	6.5	< 0.001 ^c^	
Rhinitis in the last 12 months	4.1	5.6	< 0.001 ^c^	
Burning, itchy, watery eyes	2.6	4.0	< 0.001 ^c^	
Rhinitis affected daily activities	Not at all	0.9	1.0	
	A little	2.0	2.6	
	Moderate	0.9	1.3	
	A lot	0.4	0.6	
Ever had hay fever	2.8	4.7	< 0.001 ^c^	
Itchy rash ever	0.8	1.3	< 0.001 ^c^	
itchy rash in the last 12 months	0.7	1.1	< 0.001 ^c^	
Flexural rash	0.5	1.0	< 0.001 ^c^	
Rash cleared completely	0.5	0.8	< 0.001 ^c^	
Child awakened by itchy rash in the last 12 months	Not at all	0.4	0.5	
	< Once/week	0.2	0.4	
	> Once/week	0.0	0.2	
Eczema ever	0.7	1.1	< 0.001 ^c^	

^a^ Prevalence estimates were weighted using the bootstrap weights

^b^ χ^2^ test

^c^ *P* < 0.05

Table 3 shows the prevalence and severity of asthma, allergic rhinitis, and eczema symptoms by sex. The prevalence of current wheeze, wheeze ever, wheezing limited speech to 1 or 2 words, asthma ever, exercise-induced wheeze, and nocturnal cough were significantly more common in boys than in girls (Table 3).

**Table 3:** Weighted prevalence estimates for prevalence and severity of asthma, rhinitis, and eczemain overall and by sex^a^

Asthma symptoms		Girl (*n* = 3355)	Boy (*n* = 3989)	*P*-Value^b^	
Ever had wheezing		**3.6**	**6.0**	** < 0.001**^**c**^	
Wheezing in the last 12 months(current wheeze)		**2.8**	**4.9**	** < 0.001**^**c**^	
Number of attacks of wheezing in the last 12 months	None		0.0	0.2	< 0.001 ^c^
	1–3 times		1.8	3.7	
	4–12 times		0.8	0.8	
	> 12 times		0.2	0.2	
Sleep disturbance due to wheezing in the last 12 months	None		1.0	1.8	< 0.001 ^c^
	< Once/week		1.1	2.2	
	> Once/week		0.7	0.9	
Wheezing limited speech to 1 or 2 words		**1.0**	**1.5**	** < 0.001 **^**c**^	
Asthma ever		2.0	2.7	< 0.001 ^c^	
Wheezing occurring during or after exercise		**2.1**	**3.6**	** < 0.001 **^**c**^	
Dry night cough at night unrelated to cold/chest infection		**3.9**	**5.8**	** < 0.001 **^**c**^	
Rhinitis ever		3.8	6.3	< 0.001 ^c^	
Rhinitis in the last 12 months		3.4	5.5	< 0.001 ^c^	
Burning, itchy, watery eyes		2.5	3.6	< 0.001 ^c^	
Rhinitis affected daily activities	Not at all		0.6	1.1	< 0.001 ^c^
	A little		1.5	2.7	
	Moderate		1.1	1.1	
	A lot		0.3	0.6	
Ever had hay fever		3.0	3.9	< 0.001 ^c^	
Itchy rash ever		1.1	0.9	< 0.001 ^c^	
itchy rash in the last 12 months		0.9	0.8	< 0.001 ^c^	
Flexural rash		0.7	0.7	< 0.001 ^c^	
Rash cleared completely		0.7	0.6	0.027 ^c^	
Child awakened by itchy rash in the last 12 months	Not at all		0.4	0.5	< 0.001 ^c^
	< Once/week		0.3	0.2	
	> Once/week		0.1	0.1	
Eczema ever		1.0	0.8	< 0.001 ^c^	

^a^ Prevalence estimates were weighted using the bootstrap weights

^b^ χ^2^ test

^c^ *P* < 0.05

Table 1 shows the prevalence and severity of asthma, allergic rhinitis, and eczema symptoms in the age groups of 6–7 years and 13–14 years by gender. In the age group of 6–7 years, the prevalence of current wheeze, wheeze ever, wheezing limited speech to 1 or 2 words, asthma ever, exercise-induced wheeze, and nocturnal cough were significantly more common in boys than girls. However, in the age group of 13–14 years, the prevalence of current wheeze, wheeze ever, wheezing limited speech to 1 or 2 words, asthma ever, exercise-induced wheeze, and nocturnal cough were significantly more common in girls than boys (Supplementary Table 1).

The results showed that the prevalence and severity of asthma symptoms were significantly higher in children with allergies. Because of the predominance of present wheeze, wheeze ever, and wheezing, the speech was limited to 1 or 2 words.

The prevalence of current wheeze, wheeze ever, wheezing limited speech to 1 or 2 words, asthma ever, exercise-induced wheeze, and nocturnal cough was 11(30.3 vs. 2.7), 11(37.9 vs. 3.4), 11(9.9 vs. 0.9), 12(18.6 vs. 1.6), 14 (26.3 vs. 1.9), and 10 (34.4 vs. 3.5) times, respectively, higher in children who had allergic rhinitis in the last 12 months, compared to children who did not (Supplementary Table 2).

Table 4 provide risk variables for current wheeze, their odds ratios, and 95% confidence intervals. The results of the multivariable logistic regression model showed that the variables of sex, rhinitis ever, eczema ever, history of asthma and other allergic diseases in the family, birth weight, type of childhood nutrition (breast milk, dry milk, or both), smoker(s) at home, and a pet in the child's home were significant risk factors for current wheeze. Those who had rhinitis or eczema in the past had a 12.15 and 4.88 times higher likelihood of getting current wheeze than those who did not have these diseases (Table 4).

**Table 4:** Weighted adjusted Odds Ratio (95% CI) for risk factors associated with current wheeze ^a^

Risk factor		Current wheeze		
		**OR**	**95% CI**	***P*-Value**^**b**^
Age	6–7 years	1.0	-	0.178
	13–14 years	0.96	0.91–1.02	
Sex	Girl	1.0	-	< 0.001 ^c^
	Boy	1.59	1.51–1.68	
Rhinitis ever	No	1.0	-	** < 0.001 **^**c**^
	Yes	**12.15**	**11.49–12.84**	
Eczema ever	No	1.0	-	** < 0.001 **^**c**^
	Yes	**4.88**	**4.31–5.53**	
Family history of asthma and other allergic diseases	No	1.0	-	< 0.001 ^c^
	Yes	2.22	2.11–2.35	
Birth weight	Normal	1.0	-	< 0.001 ^c^
	Low	1.60	1.40–1.82	
Breast feeding	Breast milk	1.0	-	-
	Milk Powder	1.34	1.19–1.50	< 0.001 ^c^
	Both	1.29	1.20–1.38	< 0.001 ^c^
Smoker in the household	No	1.0	-	< 0.001 ^c^
	Yes	1.32	1.24–1.42	
Pets in the home	No	1.0	-	0.001 ^c^
	Yes	1.13	1.06–1.22	

^a^ Odds Ratio (95% CI) estimates were weighted using the bootstrap weights

^b^ Weighted multivariable logistic regression model analysis

^c^ *P* < 0.05

## Discussion

It is the first research in Khuzestan Province to examine the prevalence of asthma, allergic rhinitis, and eczema in children and adolescents and some related risk factors. Both prevalence and severity of current wheeze, wheeze ever, wheezing limited speech to 1 or 2 words, asthma ever, exercise-induced wheeze, and nocturnal cough were significantly higher in boys than in girls in total and in the age group of 6–7 years. Meanwhile, in girls aged 13–14, the prevalence and severity were significantly higher than in boys.

The present study confirms the results of a meta-analysis conducted in 2013 on the prevalence of asthma among Iranian children. The prevalence of asthma among Iranian children was 3.04%.In terms of age group, the prevalence of asthma was estimated at 2.7% in children aged 6–7 years and 3.5% in adolescents aged 13–14 years [11]. However, a recently published study reported higher statistics. The prevalence of asthma in children was 6%; in adolescents, it was 8%. The prevalence was 8%-9% in girls and boys, respectively [35]. In Golestan Province, northern Iran, in 2014, Mehrvar et al. reported the overall prevalence of current asthma and asthma ever symptoms as 9.5% and 7.5%, respectively, with a higher prevalence of asthma in the age group of 13–14 years than in the age group of 6–7. In both age groups, the prevalence of asthma symptoms was higher in girls than boys [36]. Recently, a study in Karaj (near the capital of Iran) reported the prevalence of wheezing ever, current wheeze, and wheezing after exercise in adolescents aged 13–14 years 22%, 10.52%, and 22.37%, respectively [37]. In Kurdistan Province, western Iran, the overall prevalence of allergic rhinitis symptoms in children and adolescents was much higher than the results of our study. In this survey, 29.7% of children said they have sneezed or had runny noses in the past. This was significantly higher in 13–14-year-old children than in children aged 6–7 years. Regarding gender, symptoms were more common in boys in both age groups than in girls [38]. Similarly, in another study in Bushehr Province, southern Iran (2015), the prevalence of atopic eczema, allergic rhinitis, and asthma in elementary school children (6–7 years old) was 12.1%, 11.8%, and 6.7%, respectively, and secondary school students (age group 13–14 years) were 19%, 30% and 7.6%, respectively [12]. The results of the present study are consistent with the results of the mentioned studies as well as other studies conducted in Iran [16, 39, 40] regarding the higher prevalence of asthma and allergy symptoms in high school adolescents than elementary school students, which shows that the prevalence of symptoms increases with age. Air pollution is the crucial explanation for the greater prevalence in our research than in other provinces. For many years, Khuzestan Province has suffered from the air, water, and soil pollution, and sandstorms have recently worsened the situation [41–43]. According to research, exposure to dust and air pollutants such as PM_10_ increased hospital admissions and death from asthma, chronic obstructive pulmonary disease (COPD), bronchiectasis, and other respiratory disorders [44–48].

In studies on countries neighboring Iran, for example, Turkey, from April to June 2008, the prevalence of wheeze ever, current wheeze, and asthma was 13.5%, 6.3%, and 11.2% among adolescents aged 13–14, respectively [49]. In Kuwait in 2008, 2882 students aged 13–14 were randomly surveyed with the ISAAC questionnaire: the prevalence of wheeze ever was reported (13.4%), current wheeze (7.6%), allergic rhinitis ever (41.4%), current allergic rhinitis (27.6%), current itchy rash (10.6%), itchy rash ever (8.3%) [50]. Alghamdi et al. reviewed studies conducted from 1986 to 2017 in various cities in Saudi Arabia and estimated that asthma prevalence in Saudi children ranged from 8 to 25% [51]. Moreover, a review study in the Middle East conducted in 2016 estimated the overall prevalence of asthma in children in the region to be between 10 and 30 percent, with Saudi Arabia having the highest incidence (23%) while Morocco had the highest prevalence of allergic rhinitis (37.8%). The highest rate of eczema was reported for Qatar (23%) [52]. In Pakistan (2007), the prevalence of asthma in the population aged 3–16 years was 15.8%, allergic rhinitis 28.50%, and eczema 21.8% [53].

Elsewhere in the world, reports were relatively higher than the results of our study. In Gyeonggi Province, South Korea, in 2018, out of 41,062 students aged 7 to 12, the overall prevalence of asthma, allergic rhinitis, and atopic dermatitis was estimated at 5.3%, 38.4%, and 25.0%, respectively [54]. Arrais et al. (2019) in Angola surveyed 3080 children aged 6–7 years without gender segregation and estimated the prevalence of asthma symptoms at 15.8%, rhinitis at 19%, and eczema at 22% [55]. Singh et al. (2016), with a comprehensive study of Indian students, estimated the prevalence of allergic rhinitis, allergic rhinoconjunctivitis, and eczema in the age group of 6–7 years 11.3, 3.9 and 2.8%, respectively. Meanwhile, in the age group 14 to 13, these three complications were much more common and were 24.4, 10.9, and 3.7%, respectively [56]. Changes and differences in prevalence in different regions can be due to various reasons such as demographic characteristics, urbanization, altitude [57, 58], temperature [59], humidity [60], and regional differences [61].

In the present study, as in other studies [12, 15, 40, 62, 63], an association was found between the prevalence of asthma and allergic rhinitis and eczema, which confirms that the causes and risk factors for the occurrence and exacerbation of these disorders can be similar [15]. These disorders share many genetic risk variations that dysregulate the expression of immune-related genes [64]. Genetic variables account for around 90% of the variation in asthma, eczema, and rhinitis susceptibility. The remaining variation is accounted for by environmental characteristics not shared by family members [65]. Thus, the results of our study showed that in children with allergic rhinitis, the prevalence and severity of asthma symptoms are significantly higher. This is also true for children with eczema (to a lesser extent than allergic rhinitis), as the severity and prevalence of asthma symptoms are higher in children with eczema than in those without eczema. In the present study, the most common risk factors for current wheeze were rhinitis and eczema. In addition, in our study, gender was the influential risk factor. Therefore, the prevalence and severity of asthma were higher in boys. Family history of asthma and other allergic diseases, low birth weight, formula, the presence of a smoker, and the presence of a pet at home were other risk factors for this study. Allergic disorders in the family history have been reported as the most common risk factors in other studies [61, 66]. In contrast, others have reported low birth weight as one of the most influential factors [15, 67].

### Strengths and limitations

This was the first research to look at the prevalence and severity of asthma, allergic rhinitis, and eczema in children and adolescents in Khuzestan Province. The use of the primary ISAAC questionnaire, which is a trustworthy instrument for identifying the status of allergic disorders, as well as the use of correct epidemiological criteria, including the disease's typical symptoms, is another strength of our study. Another strength of this study was the size of the study region, which included many cities in Khuzestan Province with varying urban, topographical, and environmental characteristics.

However, there are several limitations to this research. Unfortunately, there is not enough longitudinal data to determine if the province's prevalence of asthma and allergy disorders has increased or decreased over time. Another drawback was the lack of study opportunities in rural schools. Because the purpose was to analyze the prevalence in industrial regions, we picked urban schools. Because this study relied on self-reported data, some bias is likely.

## Conclusions

The frequency of asthma and allergy illnesses in children in Khuzestan Province's urban regions is similar to the national average. The primary variables in this study were gender, allergic rhinitis, and eczema since the incidence and severity of asthma symptoms were higher in boys and those who had allergic rhinitis and eczema. Unfortunately, few studies have been conducted for years to examine the trend of changes in the prevalence of asthma and allergies in Khuzestan Province. Studying the prevalence of asthma and other allergic disorders in the province should be repeated over short periods of several years using proper tools and protocols such as ISAAC.

## Supplementary Information

**Additional file 1: Supplementary Table 1.** Weighted prevalence estimates for prevalence and severity of asthma, rhinitis, and eczemain 6-7 and 13-14 years old by sex^a^.

**Additional file 2: Supplementary Table 2.** Weighted prevalence estimates (%) for prevalence and severity of asthma among children with allergies^a^.

**Additional file 3. **

## Data Availability

Data sharing: Participant-level data are available from the corresponding author.

## Abbreviation

ISAAC: The International Study of Asthma and Allergies in Childhood Questionnaires
