# Supplementary material for: Assessing the prevalence and severity of asthma, rhinitis, and eczema among schoolchildren (6–7 and 13–14 years old) in Khuzestan, Iran: a cross-sectional survey
Source: BMC Pediatr. 2022 Aug 2;22:463. doi: 10.1186/s12887-022-03520-x (PMC9344770; doi:10.1186/s12887-022-03520-x)
Supplement: Supplementary file 1 — Additional file 1: Supplementary Table 1. Weighted prevalence estimates for prevalence and severity of asthma, rhinitis, and eczemain 6-7 and 13-14 years old by sexa. [file 12887_2022_3520_MOESM1_ESM.docx]

**Supplementary Table1: Weighted prevalence estimates for prevalence and severity of asthma, rhinitis, and eczemain 6-7 and 13-14 years old by sex^a^**

| **Asthma symptoms** | | **6-7 years** | | | | **13-14 years** | | | | |
| --- | --- | --- | --- | --- | --- | --- | --- | --- | --- | --- |
|  |  | **Girl (n=1806)** | **Boy(1875)** | **Total(n=3681)** | **P-Value^b^** | **Girl(n=2183)** | **Boy (n=1480)** | **Total(n=3663)** | **P-Value^b^** | |
| Ever had wheezing | | **2.5** | **6.3** | 4.5 | **<0.001^c^** | **6.4** | **5.6** | 5.9 | **<0.001 ^c^** | |
| Wheezing in the last 12 months | | **1.9** | **5.3** | 3.8 | **<0.001 ^c^** | **4.9** | **4.2** | 4.4 | **<0.001 ^c^** | |
| Number of attacks of wheezing in the last 12 months | None | 0.0 | 0.1 | 0.1 | <0.001 ^c^ | 0.1 | 0.3 | 0.3 | <0.001 ^c^ |  |
|  | 1-3 times | 1.3 | 4.6 | 3.1 |  | 3.0 | 2.5 | 2.7 |  |  |
|  | 4-12 times | 0.5 | 0.7 | 0.6 |  | 1.4 | 0.9 | 1.1 |  |  |
|  | >12 times | 0.1 | 0.0 | 0.1 |  | 0.5 | 0.4 | 0.4 |  |  |
| Sleep disturbance due to wheezing in the last 12 months | None | 0.7 | 1.8 | 1.3 | <0.001 ^c^ | 1.7 | 1.7 | 1.7 | <0.001 ^c^ |  |
|  | <Once/week | 0.8 | 2.5 | 1.7 |  | 1.9 | 1.7 | 1.8 |  |  |
|  | >Once/week | 0.4 | 1.0 | 0.7 |  | 1.3 | 0.7 | 0.9 |  |  |
| Wheezing limited speech to 1 or 2 words | | **0.6** | **1.7** | 1.2 | **<0.001 ^c^** | **1.9** | **1.3** | 1.5 | **<0.001 ^c^** | |
| Asthma ever | | 1.0 | 2.4 | 1.8 | <0.001 ^c^ | 4.3 | 3.0 | 3.4 | <0.001 ^c^ | |
| Wheezing occurring during or after exercise | | **0.8** | **3.4** | 2.2 | **<0.001 ^c^** | **5.2** | **4.0** | 4.4 | **<0.001 ^c^** | |
| Dry night cough at night unrelated to cold/chest infection | | **2.1** | **5.5** | 4.0 | **<0.001 ^c^** | **8.1** | **6.0** | 6.7 | **<0.001 ^c^** | |
| Rhinitis ever | | 2.2 | 6.6 | 4.6 | <0.001 ^c^ | 7.8 | 5.8 | 6.5 | <0.001 ^c^ | |
| Rhinitis in the last 12 months | | 1.9 | 6.0 | 4.1 | <0.001 ^c^ | 6.9 | 4.9 | 5.6 | <0.001 ^c^ | |
| Burning, itchy, watery eyes | | 1.0 | 3.9 | 2.6 | <0.001 ^c^ | 5.9 | 3.1 | 4.0 | <0.001 ^c^ | |
| Rhinitis affected daily activities | Not at all | 0.4 | 1.2 | 0.9 | <0.001 ^c^ | 1.0 | 1.0 | 1.0 | <0.001 ^c^ |  |
|  | A little | 0.8 | 3.0 | 2.0 |  | 3.1 | 2.4 | 2.6 |  |  |
|  | Moderate | 0.6 | 1.2 | 0.9 |  | 2.2 | 0.9 | 1.3 |  |  |
|  | A lot | 0.1 | 0.6 | 0.4 |  | 0.6 | 0.6 | 0.6 |  |  |
| Ever had hay fever | | 1.4 | 4.0 | 2.8 | <0.001 ^c^ | 6.6 | 3.8 | 4.7 | <0.001 ^c^ | |
| Itchy rash ever | | 1.0 | 0.6 | 0.8 | <0.001 ^c^ | 1.6 | 1.2 | 1.3 | <0.001 ^c^ | |
| itchy rash in the last 12 months | | 0.7 | 0.6 | 0.7 | <0.001 ^c^ | 1.2 | 1.0 | 1.1 | <0.001 ^c^ | |
| Flexural rash | | 0.4 | 0.5 | 0.5 | <0.001 ^c^ | 1.2 | 0.9 | 1.0 | 0.004 ^c^ | |
| Rash cleared completely | | 0.5 | 0.5 | 0.5 | <0.001 ^c^ | 1.0 | 0.6 | 0.8 | <0.001 ^c^ | |
| Child awakened by itchy rash in the last 12 months | Not at all | 0.4 | 0.5 | 0.4 | <0.001 ^c^ | 0.6 | 0.5 | 0.5 | <0.001 ^c^ |  |
|  | <Once/week | 0.3 | 0.1 | 0.2 |  | 0.5 | 0.3 | 0.4 |  |  |
|  | >Once/week | 0.1 | 0.0 | 0.0 |  | 0.1 | 0.2 | 0.2 |  |  |
| Eczema ever | | 0.8 | 0.6 | 0.7 | 0.009 ^c^ | 1.6 | 0.9 | 1.1 | <0.001 ^c^ | |

a: Prevalence estimates were weighted using the bootstrap weights.

b: χ^2^ test

c: P<0.05
