# Supplementary material for: Assessing the prevalence and severity of asthma, rhinitis, and eczema among schoolchildren (6–7 and 13–14 years old) in Khuzestan, Iran: a cross-sectional survey
Source: BMC Pediatr. 2022 Aug 2;22:463. doi: 10.1186/s12887-022-03520-x (PMC9344770; doi:10.1186/s12887-022-03520-x)
Supplement: Supplementary file 2 — Additional file 2: Supplementary Table 2. Weighted prevalence estimates (%) for prevalence and severity of asthma among children with allergiesa. [file 12887_2022_3520_MOESM2_ESM.docx]

**Supplementary Table2: Weighted prevalence estimates (%) for prevalence and severity of asthma among children with allergies ^a^**

| **Asthma symptoms** | **Rhinitis ever^b^** | | **Rhinitis in the**  **last 12 months^b^** | | **Hay fever ever^b^** | | **Eczema ever^b^** | | |
| --- | --- | --- | --- | --- | --- | --- | --- | --- | --- |
|  | **No** | **Yes** | **No** | **Yes** | **No** | **Yes** | **No** | **Yes** | |
| Ever had wheezing | 3.1 | 38.9^c^ | **3.4** | **37.9^c^** | 3.7 | 40.6 ^c^ | **4.8** | **32.2 ^c^** | |
| Wheezing in the last 12 months | 2.6 | 30.1 ^c^ | **2.7** | **30.3 ^c^** | 3.0 | 32.8 ^c^ | **3.8** | **27.8^c^** | |
| Number of attacks of wheezing in the last 12 months | None | 0.1 | 0.9 ^c^ | 0.1 | 1.0 ^c^ | 0.1 | 0.6 ^c^ | 0.1 | 3.1 ^c^ |
|  | 1-3 times | 1.9 | 21.9 | 2.0 | 21.8 | 2.1 | 23.9 | 2.8 | 17.9 |
|  | 4-12 times | 0.5 | 5.2 | 0.6 | 5.3 | 0.6 | 6.1 | 0.8 | 2.7 |
|  | >12 times | 0.1 | 2.1 | 0.1 | 2.3 | 0.1 | 2.2 | 0.2 | 4.1 |
| Sleep disturbance due to wheezing in the last 12 months | None | 0.9 | 12.2 ^c^ | 0.9 | 12.1 ^c^ | 1.1 | 10.5 ^c^ | 1.4 | 5.4 ^c^ |
|  | <Once/week | 1.2 | 10.9 | 1.3 | 11.8 | 1.3 | 13.9 | 1.7 | 11.4 |
|  | >Once/week | 0.5 | 7.0 | 0.5 | 6.4 | 0.5 | 8.4 | 0.7 | 11.1 |
| Wheezing limited speech to 1 or 2 words | 0.8 | 9.8 ^c^ | **0.9** | **9.9^c^** | 0.9 | 12.2^c^ | **1.2** | **11.1^c^** | |
| Asthma ever | 1.5 | 19.1 ^c^ | 1.6 | 18.6 ^c^ | 1.6 | 23.5 ^c^ | **2.2** | **23.4^c^** | |
| Wheezing occurring during or after exercise | 1.7 | 26.8 ^c^ | **1.9** | **26.3^c^** | 1.9 | 32.2 ^c^ | **2.8** | **27.5^c^** | |
| Dry night cough at night unrelated to cold/chest infection | 3.3 | 35.1 ^c^ | **3.5** | **34.4^c^** | 3.6 | 41.8 ^c^ | **4.7** | **41.8^c^** | |

a: Prevalence estimates were weighted using the bootstrap weights.

b: χ^2^ test

c: P<0.05
