# Supplementary material for: Assessing the prevalence and severity of asthma, rhinitis, and eczema among schoolchildren (6–7 and 13–14 years old) in Khuzestan, Iran: a cross-sectional survey
Source: BMC Pediatr. 2022 Aug 2;22:463. doi: 10.1186/s12887-022-03520-x (PMC9344770; doi:10.1186/s12887-022-03520-x)
Supplement: Supplementary file 3 — Additional file 3. [file 12887_2022_3520_MOESM3_ESM.docx]

Supplementary-1

***Sampling***

Sampling was carried out in the following eight steps:

1- Khuzestan province's whole school society (seen as clusters) was separated into two strata: elementary and secondary schools.

2- Because the entire number of primary and secondary schools (total clusters) in Khuzestan province was almost equal, it was decided to choose 100 schools (clusters) in each class (elementary and middle) to get a sample of 4,000 students and 40 students from each school.

3- Seven inhabited cities in Khuzestan province were found in five geographical directions by developing a geographical map of the province (north, south, east, west and center)

4- Probability Proportional to Size was used to determine the number of schools (clusters) necessary for sampling in each city (PPS). So, in each city, the number of clusters equals the number of schools in that city divided by the total number of schools in the province, multiplied by 100 (initial number of clusters in province).

5- Then, in each city, a list of clusters (primary and secondary schools) was created, together with the population of students in each cluster, and clusters were organized depending on the population of their pupils.

6- The sample distance in each city was computed by dividing the total population of clusters by the number of necessary clusters (the number of necessary clusters from each city was obtained in step 3).

7- Cluster sampling was done using systematic random sampling in each city.

8- In step 6, a sample of 40 students was gathered from each identified cluster. If a cluster had fewer than 40 students, the closest cluster was also considered. In the age group of 6-7 years, the questionnaires were completed by the parents of the children and in the age group of 13-14 years by the children themselves.
